# Neuromodulatory effects of N-acetyl-L-leucine in a human induced neuronal cell culture system

**DOI:** 10.3389/fimmu.2026.1866462

**Published:** 2026-08-05

**Authors:** Adriana Rehm, Khadija Boulahyoun, Luisa Klossek, Pia Renk, Tom Pietruschka, Ralf Gold, Jeremias Motte, Anke Salmen

**Affiliations:** Department of Neurology, Center for Clinical Research, St. Josef-Hospital, Ruhr University Bochum, Bochum, Germany

**Keywords:** cell culture, hiPNs, multiple sclerosis, NALL, neuroprotection, oxidative stress

## Abstract

N-acetyl-L-leucine (NALL), an acetylated derivative of the amino acid leucine, has been shown to reduce neuronal cell death and neuroinflammation in murine models. Its beneficial effects in patients with the lysosomal storage disease Niemann-Pick Type C have led to recent FDA and EMA approval. However, neuroprotective effects of NALL remain to be further elucidated. In this study, we investigate and characterize the neuroprotective effects of NALL. To this end, we used human induced primary neurons (hiPNs), which were generated from induced pluripotent stem cells derived from reprogrammed renal proximal tubule epithelial cells obtained from either healthy controls (HC) or individuals with relapsing-remitting multiple sclerosis (MS). We demonstrated that NALL exhibits neuroprotective properties in MS- and HC-derived hiPNs subjected to acute damage induced by the microtubule-destabilizing agent nocodazole determined by neurite length. This effect can be blocked by inhibition of transporter-specific NALL uptake. HiPNs from MS donors treated with NALL expressed higher levels of glutamate cysteine ligase (GCL), the ratelimiting enzyme in glutathione synthesis. MS-specific cells are more susceptible to stress induced by the protein kinase inhibitor staurosporine, whereas in HC-specific cells only, NALL is able to modulate this stress induction. In summary, we demonstrate differential responses to induced stress in MS- and HC-specific neurons and the capacity of NALL to modulate neurite outgrowth and stress mechanisms in this cell culture system. NALL may represent an interesting additive treatment for MS or other diseases associated with oxidative stress and neurodegeneration if further substantiated.

## 1 Introduction

Multiple sclerosis (MS) is a chronic inflammatory disease of the central nervous system (CNS) mostly affecting young adults, particularly women (female-to-male ratio up to 3:1) (1–3). MS is characterized by autoimmune processes in the CNS causing chronic inflammation, demyelination and neurodegeneration leading to impaired neuronal signal conduction and progressive neurological dysfunction. The activation of microglia and the infiltration of immune cells lead to the sustained production of reactive oxygen species (ROS) and reactive nitrogen species, which damage molecules such as lipids, proteins, and nucleic acids, causing cellular injury and mitochondrial dysfunction. This dysfunction leads to higher ROS levels, amplifying oxidative stress, and contributes to neurodegeneration and progressive damage, independently of acute inflammatory processes (4–7).

While relapsing forms of MS (RMS) respond relatively well to modern treatment targeting mainly the immune-mediated parts of the pathophysiology, treatment options for progressive forms are rather limited (8, 9). In RMS, focal inflammatory activity seems to be the predominant driver of acute damage, whereas progressive forms are more degenerative in nature and do not predominantly involve focal inflammation, but subtle CNS-inherent inflammation involving innate immune cells (9, 10). However, current evidence suggests that MS is a disease continuum and in all disease stages, even very early on, neurodegenerative mechanisms driving progression independent of relapses (PIRA) run in parallel to the inflammatory processes (11, 12). Therefore, complementary therapeutic approaches that target mechanisms beyond acute inflammation, including neurodegeneration and oxidative stress, represent an unmet need.

As an acetylated derivative of leucine, N-acetyl-leucine (NAL) improves neurological functions in cerebellar ataxias (13, 14). The racemate N-acetyl-DL-leucine has long been used as a medication for treating acute vertigo and vertiginous symptoms in France (15, 16). Electrophysiological studies on guinea pigs have revealed that DL-NAL can restore the membrane potential of abnormally polarized neurons in the brain (17).

It has been demonstrated that N-Acetyl-L-leucine (NALL) is driving therapeutic effects of NAL. Studies with rats have consecutively shown that NALL improves central compensation of postural symptoms in acute vestibular lesions (18). In studies using a mouse model of Niemann-Pick disease type C (a neurodegenerative lysosomal storage disorder), NALL was identified having neuroprotective and disease-modifying effects (19). In a mouse model with traumatic brain injury, NALL improves neurological function and restricts neuronal loss and neuroinflammation after injury (16). In clinical trials with patients with Niemann-Pick disease Type C and ataxia-teleangiectasia, improved ataxic symptoms and stabilized disease progression were observed after NALL treatment (20–22).

These observations make NALL an attractive substrate within the context of MS. However, the neuroprotective effects of NALL in context of MS remain to be elucidated.

## 2 Materials and methods

### 2.1 Cohort and ethical statement

For the establishment of the human induced primary neuron (hiPN) cell culture, urine samples of people with RMS (MS) from St. Josef-Hospital Bochum, University Hospital, and healthy controls (HC) were recruited. This study was approved by the ethics committee of the Faculty of Medicine, Ruhr-University Bochum (Reg. no. 4493-12; 22-7563-NIS), and conducted in accordance with 1964 Declaration of Helsinki and its later amendments. All participants provided written informed consent. Every experiment was carried out on both HC- and MS-specific hiPNs. Details of the participants are given in **Supplementary Table 1**.

### 2.2 Human induced primary neuron model

HiPNs were differentiated as previously described (23, 24). Renal proximal tubule epithelial cells (RPTECs) from people with MS and HC were isolated from urine samples and cultivated in Renal Epithelial Cell Growth Medium™ from Lonza (#CC-3190). After a few days, RPTECs proliferate in growing colonies. After passaging RPTECs for three times, cells were reprogrammed via electroporation with the Neon^®^ Transfection system (Life Technologies) and the episomal plasmids carrying the pluripotency genes pCXLE-hOCT3/4-shp53 (#27077), pCXLE-hUL (#27080) and pCXLE-hSK (#27078) (all from Addgene) due to manufacturer’s instructions. Afterwards transfected cells were cultivated in TeSR^EM^-E7^EM^ medium (#05914, STEMCELL Technologies) until induced pluripotent stem cell (iPSC) colonies developed. IPSCs were then transferred to mTeSR™-1 (#85850, STEMCELL Technologies) medium for further cultivation. After four times passaging iPSCs were used for embryoid body (EB) formation. Therefore, iPSC colonies were scratched and cultivated under non-adherent conditions in mTeSR™-1 medium. In this stage of differentiation, medium was supplemented with 10µM SB431542 (#FBM-10-2443, BIOZOL) and 5µM dorsomorphin (#866405-64-3, Sigma) to inhibit the formation of mesoderm and entoderm with a medium change and fresh supplementation every second day. Six days after EB cultivation in free-floating conditions, EBs were replated onto a 0.002% poly-L-ornithine (#27378-49-0, Sigma) and 10µg/mL laminin (#114956-81-9, Sigma)- coated cell culture dish. For the EB cultivation under adherent conditions, medium was changed to neural stem cell (NSC) medium containing Dulbecco’s modified Eagle’s medium (DMEM)/F12 GlutaMAX™ (#31331028, Thermo Fisher Scientific), 20µg/mL insulin (#91077C, Sigma), 1.6 g/L L-glucose (AppliChem), 1µL/mL B27™ supplement (#17504-044, Life Technologies), 10 µL/mL N-2 supplement (17502048, Life Technologies) supplemented with 10 ng/mL epidermal growth factor (EGF) (#1101001, PAN Biotech) and 10 ng/mL basic fibroblast growth factor (bFGF) (#1102021, PAN Biotech). Between day seven and twelve of adherent cultivation conditions, EBs form neural rosettes. These structures were isolated and trypsinized for replatation in NSC medium. The isolated and replated cells are neural precursor cells (NPCs). For differentiation of NPCs to hiPNs cells were trypsinized and transferred onto poly-L-ornithine/laminin-coated cell culture dishes with 200,000 cells per dish. Therefore, NPC from passage 10–35 were used. On the next day, medium was changed from NSC medium to neuronal differentiation medium containing DMEM/F12 GlutaMAX™, 20 µL/mL N-2 supplement, 40 µL/mL B-27™ supplement, 50 µg/mL apo-transferrin (#T8158, Sigma) and 200 µg/mL L-ascorbic acid (#3525.1, Carl Roth) supplemented with 500 ng/mL sonic hedgehog (#100-45, PeproTech) and 4 µM retinoic acid (#R2625, Sigma) until day seven. On day seven supplementation changed to 10 ng/mL brain-derived neurotrophic factor (BDNF) (#450-02, PeproTech) and 20 ng/mL glial cell-derived neurotrophic factor (GDNF) (#450-10, Peprotech) until day 21. If not otherwise stated, cells were cultured at 37 °C and 5% CO_2_ during the whole differentiation period. A figure summarizing the generation of hiPNs and immunocytochemistry for neuron-specific marker expression and negative control stainings are given in **Supplementary Figures 1** and **2**.

### 2.3 Investigation of cell viability and apoptosis

For these experiments, hiPNs were seeded onto a poly-L-ornithine/Laminin coated BRAND^®^ 96 well, flat bottom, white microplate (BRAND GmbH+ Co. KG, Wertheim, Germany, supplied by MERCK) in a density of 45.000 cells per well.

The Caspase-Glo^®^ 3/7 assay (Promega Corporation, Madison, WI, US) was performed to investigate apoptosis in hiPNs. HiPNs were therefore either treated with staurosporine (Enzo life science, Farmingdale US) (ST; 1 µM), a protein kinase inhibitor, alone or ST (1 µM) and NALL (1 mM or 5 mM kindly provided by IntraBio, Texas, US) simultaneously for 6 or 24 hours.

The CellTiter-Glo^®^ 2.0 assay (Promega Corporation, Madison, WI, USA) was performed to investigate cellular metabolism by quantifying adenosine triphosphate (ATP), which indicates the presence of metabolically active cells. HiPNs were treated with NALL only (1 mM or 5 mM) for 30 minutes, 1 hour, 6 hours and 24 hours.

Additionally, hiPNs were either treated with ST (1 µM) alone or received a pretreatment with NALL (1 mM or 5 mM) for 24 hours and were subsequently exposed to ST (1 µM) and NALL (1 mM or 5 mM) simultaneously for 6 hours. The ST alone condition did not receive any pretreatment.

Both assays were performed according to the manufacturers’ instructions and normalized to the respective control condition. Measurements were conducted using the CLARIOstar Plus microplate reader (BMG LABTECH GmbH, Ortenberg, Germany).

### 2.4 Observation of neuroregenerative and -protective capacities

For these experiments 55.000 hiPNs per well were plated onto a poly-L-ornithine/Laminin coated 24 well plate.

To investigate whether NALL itself has an impact on neurites, HC- and MS-specific hiPNs were treated with NALL (1 mM and 5 mM) for 24 hours.

Consecutively, two different experiments were conducted to analyze neuroregenerative and -protective capacities of NALL.

To analyze regeneration capacity, hiPNs were treated with the microtubule-destabilizing reagent nocodazole (Calbiochem, Darmstadt, Germany) (noco, 10 µM) for 6 hours. The medium was then changed, followed by a 24-hour recovery phase with and without NALL treatment (1 mM and 5 mM).

To analyze neuroprotective effects, hiPNs were treated noco (10 µM) or a noco/NALL cotreatment at concentrations of 1 mM and 5 mM for 6 hours, after which they were fixed with ice-cold methanol and stained with ß-III tubulin and DAPI.

Evaluation was performed by measuring neurite length using the NeuronJ plugin of ImageJ. The sum length of all neurites measured in an image was divided by the number of cell nuclei counted to calculate the average neurite length.

### 2.5 Investigation of transporter-specific uptake of NALL

For the NALL uptake analysis 60.000 hiPNs per well were plated onto a poly-L-ornithine/Laminin coated 24 well plate.

To investigate and specify the uptake of NALL via the monocarboxylate transporter 1 (MCT1), hiPNs were pretreated with AZD3965 (MedChemExpress, NJ, US) (AZD; 20 µM and 50 µM), blocking MCT1, for 24 hours. After that, hiPNs were treated with noco (10 µM) alone or continued AZD (20 µM and 50 µM)/noco (10 µM)/NALL (1 mM and 5 mM) simultaneously for 6 hours. After fixation with ice-cold methanol, hiPNs were stained with ß-III tubulin and DAPI.

Evaluation of neurite length was performed with NeuronJ plugin of ImageJ as described above.

### 2.6 Quantitative real time PCR: oxidative stress and pathway analyses

For qRT-PCR analysis, RNA was isolated using the RNeasy Mini Kit from Qiagen (QIAGEN, Aarhus, Denmark) following manufacturers’ instructions and measured with a Nanodrop ND-1000 spectrophotometer (Implen, GmbH, Munich, Germany) based on the Beer and Lambert-Bougert-Law. CDNA was generated with the qScript™ cDNA Supermix from Promega (Promega Corporation, Madison, WI, US) according to manufacturers’ protocol. QRT-PCR was performed with the QuantStudio™ 3 (Thermo Fisher Scientific, Foster City, CA, US) by using the GoTaq^®^ qPCR master mix SYBR green (Promega Corporation, Madison, WI, US) in duplicates.

Two different experimental setups were performed to analyze oxidative stress markers and pathway analysis: First, the cells were treated with NALL (1 mM and 5 mM) for 24 hours to determine which effects NALL itself has on hiPNs. Secondly, protection against oxidative stress was analyzed. Therefore, hiPNs were treated with ST (1 µM) and 1 mM or 5 mM NALL simultaneously for 6 hours. To precondition them prior to exposure to an oxidative stressor we also investigated a pretreatment with NALL for 24 hours (1 mM or 5 mM) with a subsequent cotreatment of ST (1 µM) and NALL (1 mM or 5 mM) for 6 hours.

The following targets were investigated in terms of oxidative stress: glutamate-cysteine ligase (GCL); nuclear factor (erythroid-derived 2)-like 1 (NRF2); NAD(P)H:quinone oxidoreductase 1 (NQO1); and glutathione reductase (GSR).

To understand potential pathway mechanisms of NALL in hiPNs, analysis of primary pathways was performed under the same conditions in which oxidative stress targets were analyzed. Main pathway targets were mechanistic target of rapamycin (mTOR), proteinkinase B (AKT3), and mitogen-activated protein kinase 1 (MAPK1) as well as the involvement of the B-cell lymphoma 2 (BCL2), BCL2 antagonist on cell death (BAD) and BCL2 associated X protein (BAX), all targets of the BCL2 family.

Data were analyzed with the Pfaffl analysis method (25). The mean CT was used for analysis and housekeeping genes (TATA-Box binding protein (TBP) and glyceraldehyde-3-phosphate dehydrogenase (GAPDH)) were used to normalize target gene expression levels.

### 2.7 Statistics

Statistical analyses were performed using GraphPad Prism 10 (GraphPad Prism version 10.6.1, GraphPad Software, Boston, Massachusettes, US). All data are presented as the mean ± standard deviation (SD). The applied statistical test and the number of biological (N) and technical (n) replicates are provided in the respective figure legends. Group comparisons were always performed in the same order to limit the total number of tests: In experiments without induced damage, all treatment groups were compared to the normalized control condition. In experiments with induced damage, the damage group was compared to the normalized control condition and the treatment groups were compared to the damage condition. Only non-parametric tests were applied. For comparison of two groups Mann-Whitney test was performed. For comparisons involving more than two groups, the Kruskal–Wallis test with Dunn’s *post-hoc* test was used. Correction for multiple testing was performed within each experimental set as done by the respective *post-hoc* test applied for comparison of more than two groups. Other than that, no correction for multiple testing was performed for these exploratory analyses. P values of <0.05 were thus considered significant within each experimental section.

## 3 Results

### 3.1 NALL has no effects on functional assays of apoptosis and viability of hiPNs

In the first part of the study, we wanted to examine the functional effects of NALL on hiPNs and investigated caspase 3/7 activity and ATP activity.

**Figure 1:**
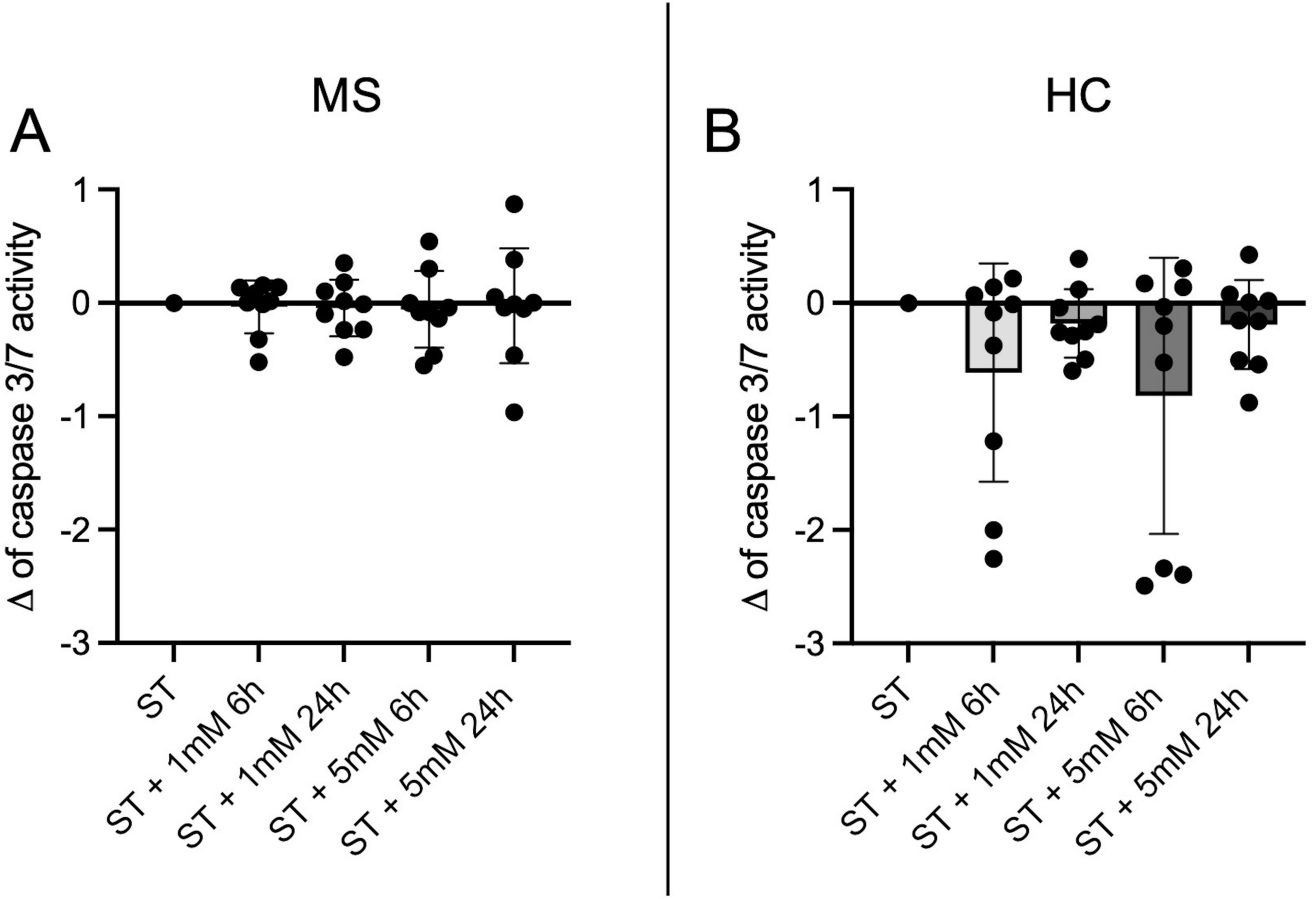
Caspase 3/7 analysis of hiPNs reveals no significant changes of ST/NALL cotreatment compared to ST alone. HiPNs from MS **(A)** or HC **(B)** were treated with ST/NALL simultaneously over 6 or 24 hours. Data represented as mean ± SD differences (Δ) to ST. Statistics: Kruskal-Wallis with Dunn’s *post-hoc* test (N = 3, n=9). HC, healthy control; hiPNs, human induced primary neurons; h, hour; MS, multiple sclerosis; NALL, N-acetyl-L-leucine; ST, staurosporine.

Caspases 3 and 7 belong to the family of executioner caspases and mediate the final steps of apoptosis by cleaving essential cellular components. Their activation is commonly used as an indicator of programmed cell death (26). Caspase 3/7 activity was observed by treating hiPNs with ST or ST/NALL simultaneously for 6 or 24 hours (h) in MS- and HC-specific hiPNs. Basal caspase 3/7 activity after ST-induced stress was significantly upregulated in MS- and HC-specific hiPNs (ST-induced fold change relative to control 1.74±0.66; p= 0.0028). No significant group differences were observed between the ST and ST/NALL groups (MS: ST + 1 mM NALL 6h -0.04±0.23; ST + 1 mM NALL 24h - 0.05±0.25; ST + 5 mM NALL -0.06±0.34; ST + 5 mM NALL 24h -0.03±0.51; each p= ns; **Figure 1A**; HC: ST + 1 mM NALL 6h - 0.61±0.96; ST + 1 mM 24h NALL -0.18±0.30; ST + 5 mM NALL 6h - 0.82±1.22; ST + 5 mM NALL 24h -0.19±0.39; each p= ns; **Figure 1B**).

**Figure 2:**
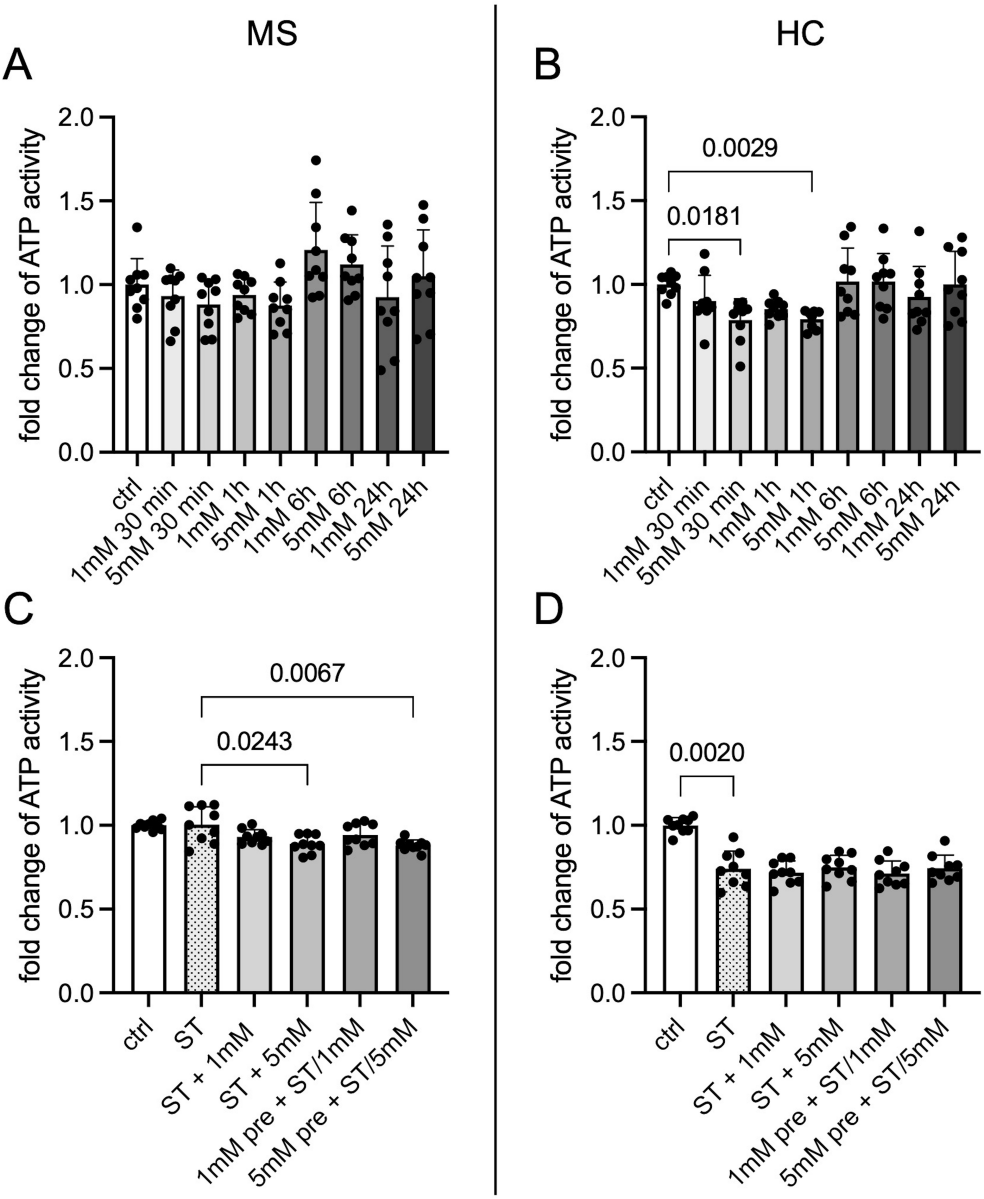
Analysis of ATP activity of hiPNs. In **(A, B)** hiPNs were treated with NALL for 30 minutes, 1 hour, 6 hours and 24 hours. **(A)** MS-specific hiPNs reveal no significant changes in ATP activity after NALL treatment. **(B)** HC-specific hiPNs showed significantly downregulated ATP levels after 5 mM NALL treatment for 30 minutes and 1 hour. **(C)** In MS-specific hiPNs treatment with ST and NALL simultaneously and with ST/NALL after 24-hour NALL pretreatment showed significantly downregulated ATP activity only at the 5 mM NALL concentration compared to ST treatment alone. **(D)** In HC-specific hiPNs, ST reduced ATP activity, but NALL treatment reveals no additional effects on ATP activity. Data represented as mean ± SD. Statistics: Kruskal-Wallis with Dunn’s *post-hoc* test (N = 3, n=9). ATP, adenosine triphosphate; HC, healthy control; hiPNs, human induced primary neurons; h, hour; MS, multiple sclerosis; NALL, N-acetyl-L-leucine; ST, staurosporine.

Cell viability was assessed using ATP measurements. ATP is the primary energy molecule, reflecting the metabolic state and viability of hiPNs. Changes in ATP levels provide an opportunity to analyze the metabolic state and behaviour of hiPNs under different conditions (27). We treated hiPNs with NALL for 30 minutes, 1 hour, 6 hours or 24 hours (**Figures 2A, B**). Significant downregulation of ATP activity was observed after 30 minutes and 1 hour of 5 mM NALL treatment in HC-specific hiPNs only (fold change of ATP activity to control: 5 mM NALL 30 minutes 0.79±0.13; p= 0.0181; 5 mM NALL 1 hour 0.79±0.06; p= 0.0029; **Figure 2B**). Significant changes were neither observed at any other time points nor in MS-specific hiPNs (each p= ns; **Figures 2A, B**).

To investigate how ATP levels differ under ST treatment, we expanded our experiments into two different setups. One group of hiPNs was treated with ST/NALL for 6 hours, while the other received a 24-hour pretreatment with NALL, followed by ST/NALL treatment for 6 hours (**Figures 2C, D**). In MS-specific hiPNs, ST alone did not significantly impact ATP levels (**Figure 2C**). We detected significantly reduced ATP levels in the ST/NALL 5 mM condition, both with and without pretreatment, although of small extent (ST + 5 mM NALL 0.89±0.05; p= 0.0243; pre NALL/ST/5 mM NALL 0.88±0.03; p= 0.0067; **Figure 2C**). ST alone resulted in reduced ATP levels only in HC-specific hiPNs (ST 0.74±0.12; p= 0.0020; **Figure 2D**). No significant changes were observed in the HC-specific hiPNs with regards to NALL exposure (each p= ns; **Figure 2D**).

### 3.2 NALL reveals neuroprotective but no neuroregenerative effects in neurite length of hiPNs

We investigated the neuroprotective and neuroregenerative effects of NALL using morphological assays via neurite length measurements. Neither positive nor negative effects on neurite length were observed with sole NALL treatment (1 mM or 5 mM) over 24 hours (absolute neurite length in µM; MS: control 100.5±12.54; 1 mM NALL 115.1±22.65; 5 mM NALL 107.2±32.87; HC: control 105.1±28.53; 1 mM NALL 95.09±17.57; 5 mM NALL 83.21±13.93; each p= ns) as a prerequisite.

**Figure 3:**
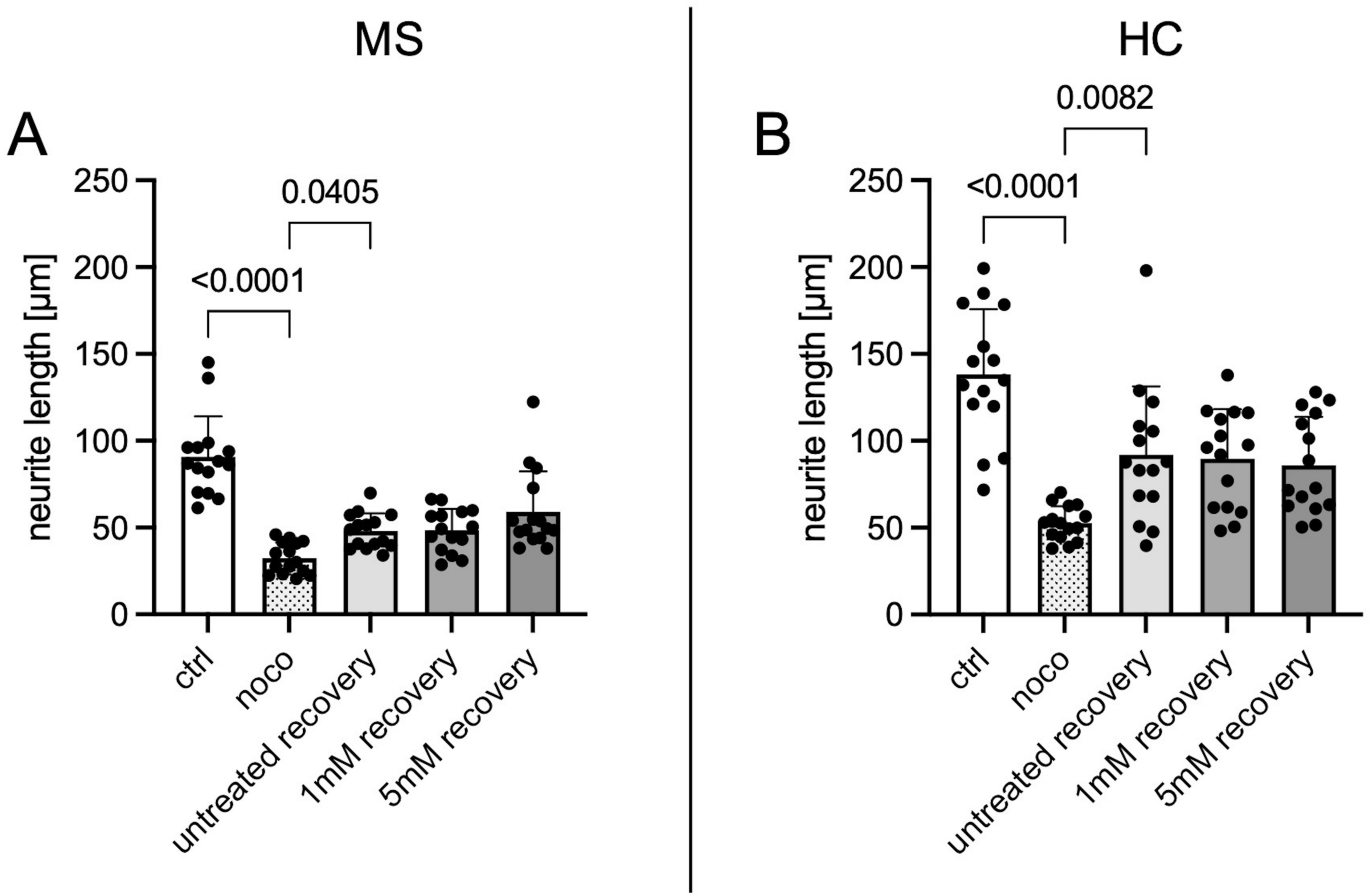
Neurite length measurements of hiPNs after damage with noco for 6 hours and 24-hour recovery phase with or without NALL. **(A)** MS-specific hiPNs showed significant damage of neurites after noco treatment and a significant recovery of neurites after 24 hours irrespective of NALL. **(B)** HC-specific hiPNs showed the same results. Data represented as mean ± SD. Statistics: Kruskal-Wallis with Dunn’s *post-hoc* test (N = 3, n=15). ctrl, control; HC, healthy control; hiPNs, human induced primary neurons; MS, multiple sclerosis; NALL, N-acetyl-L-leucine; noco, nocodazole.

To examine neuroregenerative effects, hiPNs were treated with noco for 6 hours, after which they were allowed to recover for 24 hours, either with or without NALL. Significant damage to hiPNs was observed with noco treatment (MS: control 90.80±23.32; noco 32.35±8.98; p= <0.0001; HC: control 138.2±37.63; noco 52.50±9.89; p= <0.0001; **Figures 3A, B**), as well as significant recovery in the untreated recovery condition (MS: 48.07±10.19; p= 0.0405; HC: 91.97±39.44; p= 0.0082; **Figures 3A, B**). There were no significant differences in the recovery ability of hiPNs treated with NALL compared to the untreated recovery after the recovery phase (MS: 1 mM NALL recovery 48.49±12.26; 5 mM NALL recovery 59.16±23.14; HC: 1 mM NALL recovery 89.78±28.34; 5 mM NALL recovery 85.93±27.94; each p= ns; **Figures 3A, B**). A formal comparison of MS- and HC-specific neurites was not performed.

**Figure 4:**
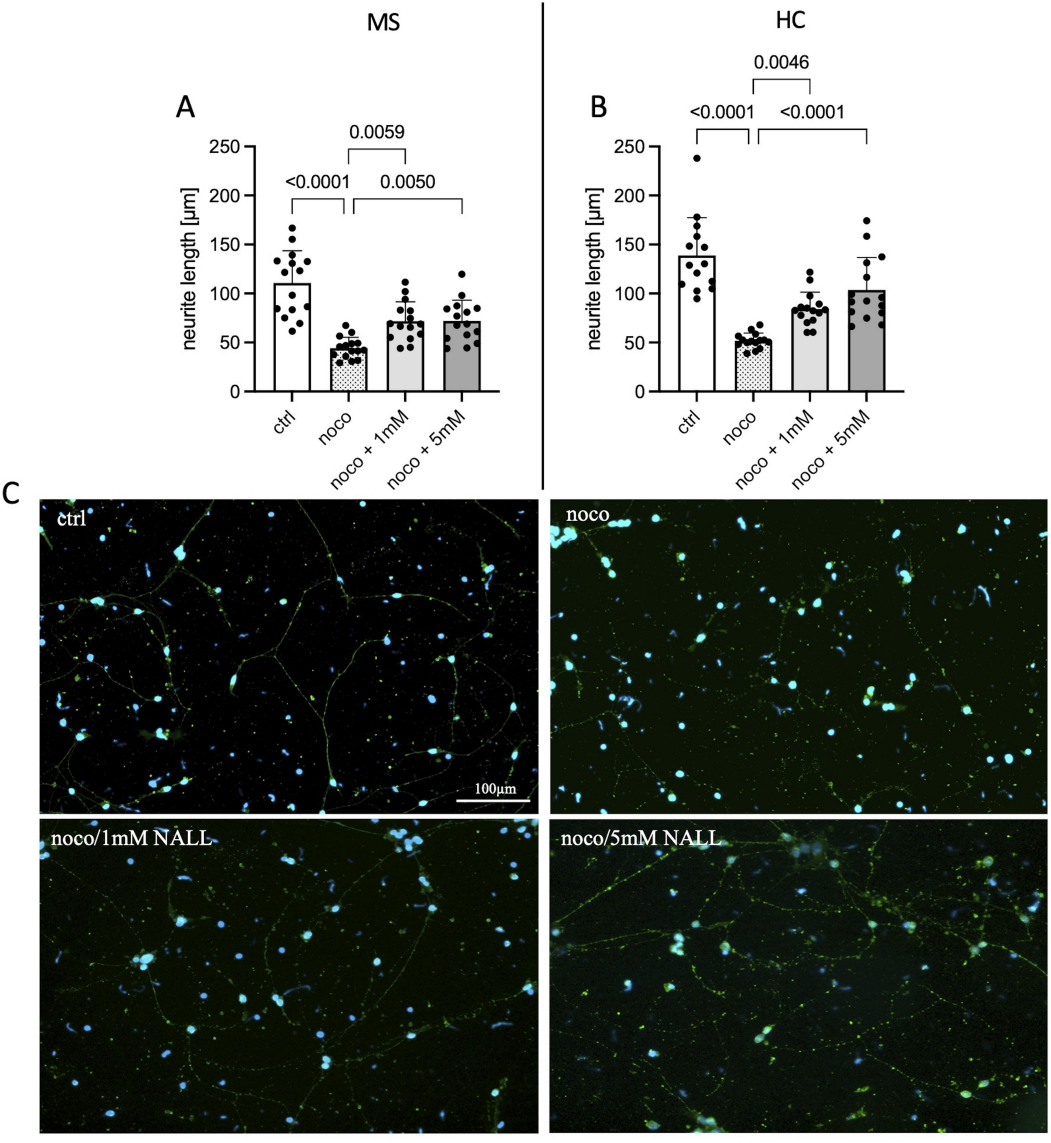
Neurite length measurements after cotreatment with noco/NALL for 6 hours. **(A)** Significantly shorter neurites were detected after noco treatment alone. Significantly longer neurites were detected after 6-hour noco/NALL cotreatment at both dosages in MS-specific hiPNs. **(B)** HC-specific hiPNs reveal the same results. **(C)** Exemplary histological representation of neurites in HC-specific hiPNs using ßIII-tubulin staining for neurites (green) and DAPI for cell nucleus (blue): upper left panel: control condition, upper right panel: noco condition, lower left panel: noco and NALL 1 mM cotreatment, lower right panel: noco and NALL 5 mM cotreatment. Scale bar: 100µm. Data represented as mean ± SD. Statistics: Kruskal-Wallis with Dunn’s *post-hoc* test (N = 3, n=15). ctrl, control; DAPI , 4′,6-Diamidino-2-phenylindol; HC, healthy control; hiPNs, human induced primary neurons; MS, multiple sclerosis; NALL, N-acetyl-L-leucine; noco, nocodazole.

To investigate potential neuroprotective mechanisms of NALL during damage, experiments involved the simultaneous administration of NALL and noco for 6 hours. Here, hiPNs receiving cotreatment exhibited significantly longer neurite lengths compared to those receiving noco treatment alone in both MS and HC conditions (MS: control 110.8±32.91; noco 44.37±11.00; p= <0.0001; noco/1 mM NALL 71.69±19.92; p= 0.0059; noco/5 mM NALL 71.96±21.30; p= 0.0050; **Figure 4A**; HC: control 138.8±38.52; p= <0.0001; noco 52.85±7.77; p= <0.0001 noco/1 mM NALL 84.30±16.95; p= 0.0046; noco/5 mM NALL 103.7±32.95; p= <0.0001; **Figure 4B**).

In summary, NALL was thus not capable of enhancing recovery after a damaging event, but elicited protective effects when present during nocodazole damage.

### 3.3 NALL effects can be reversed by blocking MCT1

In a next step, we aimed to investigate whether neuroprotective effects of NALL can be reversed when blocking the NALL uptake by MCT1 as its described transmembrane transporter (15).

**Figure 5:**
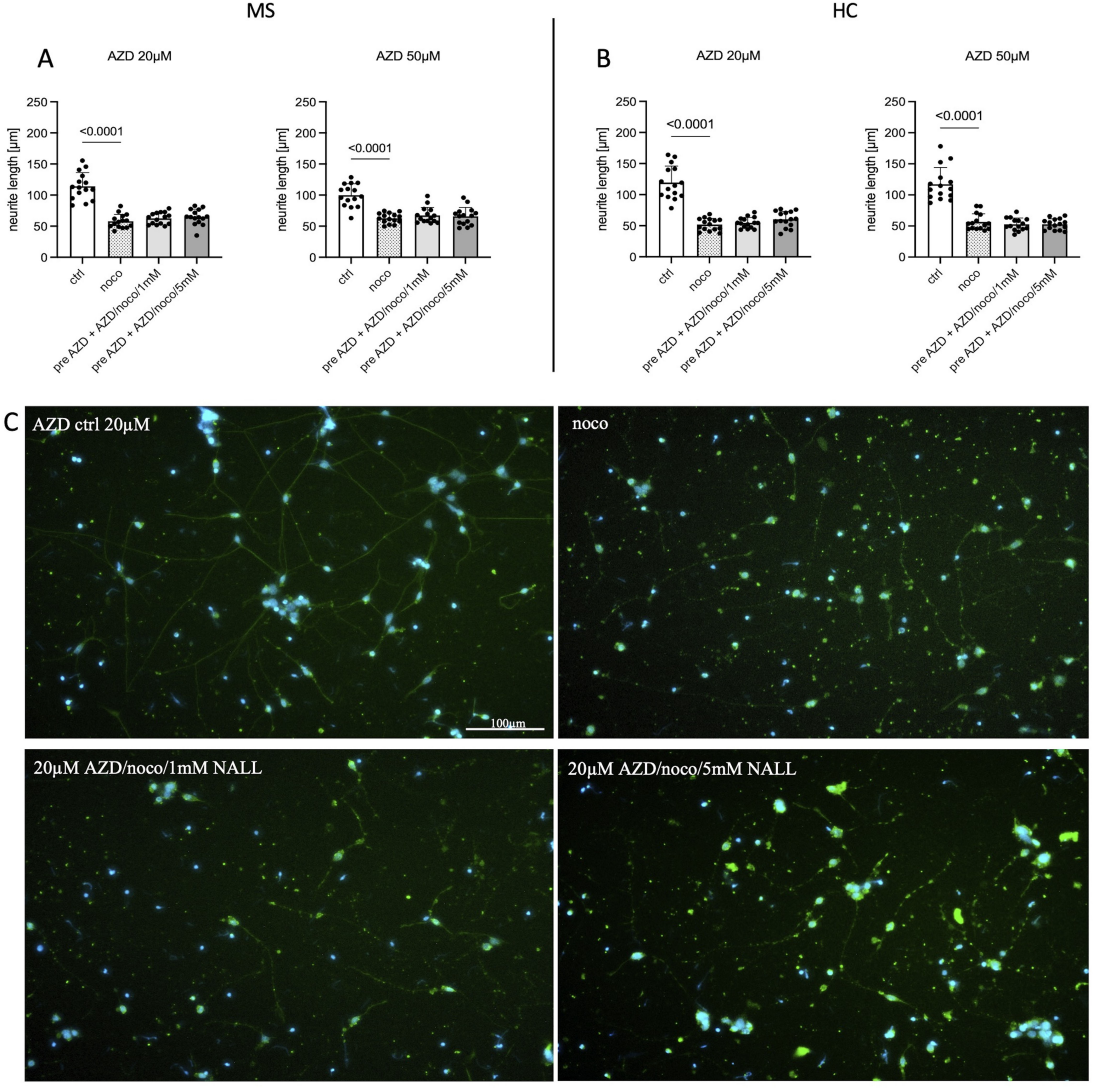
NALL effects are reversed under MCT1 blockade. MS-specific hiPNs **(A)** and HC-specific hiPNs **(B)** were pretreated with AZD (20 µM or 50 µM) for 24 hours and subsequently treated with AZD/noco/NALL for 6 hours. Noco damage to neurites was effective, but both dosages of AZD (20 µM and 50 µM) prevented neurite outgrowth promoted by both NALL concentrations in MS and HC. **(C)** Exemplary histological representation of neurites in MS-specific hiPNs using ßIII-tubulin staining for neurites (green) and DAPI for cell nucleus (blue): upper left panel: control condition, upper right panel: noco condition, lower left panel: 20µM AZD/noco/1 mM NALL, lower right panel: 20 µM AZD/noco/5 mM NALL. Scale bar: 100µm. Data represented as mean ± SD. Statistics: Kruskal-Wallis with Dunn’s *post-hoc* test (N = 3, n=15). ctrl, control; DAPI , 4′,6-Diamidino-2-phenylindol; HC, healthy control; hiPNs, human induced primary neurons; MS, multiple sclerosis; NALL, N-acetyl-L-leucine; noco, nocodazole.

To this end, we pretreated MS- and HC-specific hiPNs with 20 µM and 50 µM of AZD (MCT1 inhibitor) for 24 hours, after which we treated them with AZD/noco/NALL cotreatment for 6 hours. Contrary to our previous findings of significantly higher neurite lengths during noco/NALL cotreatment (**Figure 4**), we observed no significant changes in neurite length in the NALL-containing conditions when blocking MCT1 by AZD in hiPNs (MS: 20 µM AZD: control 114.4±21.80; noco 57.95±11.15; p= <0.0001; pre AZD+ AZD/noco/1 mM NALL 62.52±9.35; pre AZD+AZD/noco/5 mM NALL 64.82±12.50; each p= ns; **Figures 5A, C**; 50 µM AZD: control 100.0±18.23; noco 62.92±8.16; p= <0.0001; pre AZD+ AZD/noco/1 mM NALL 67.41±12.88; pre AZD+AZD/noco/5 mM NALL 65.94±14.30; each p= ns; **Figure 5A**. HC: 20 µM AZD: control 119.4±26.59; noco 52.00±9.814; p= <0.0001; pre AZD+ AZD/noco/1 mM NALL 54.74±8.580; pre AZD+AZD/noco/5 mM NALL 60.58±12.69; each p= ns; 50 µM AZD: control 117.2±26.82; noco 56.47±13.30; p= <0.0001; pre AZD+ AZD/noco/1 mM NALL 52.82±10.05; pre AZD+AZD/noco/5 mM NALL 52.79±8.812; each p= ns; **Figure 5B**).

### 3.4 MS-specific hiPNs treated with NALL have more of the ratelimiting enzyme GCL

The third part of the study examined the behaviour of hiPNs in relation to oxidative stress.

The NRF2 signaling pathway is a key regulator of cellular response to oxidative stress. When oxidative stress is induced, NRF2 translocates into the nucleus and binds to antioxidant response elements (AREs) and enzymes like NQO1 (28, 29). NQO1 as an antioxidative enzyme can reduce quinones and limit the production of ROS (30, 31). GCL is the ratelimiting enzyme that plays a pivotal role in the production of glutathione, the most essential antioxidant in cells (32). GSR maintains cellular redox homeostasis by reducing glutathione from its oxidized form GSSG. Together, these targets help cells to develop greater defense mechanisms against oxidative stress and potential stressors. To determine expression of these targets in the context of NALL treatment, we examined different experimental settings.

**Figure 6:**
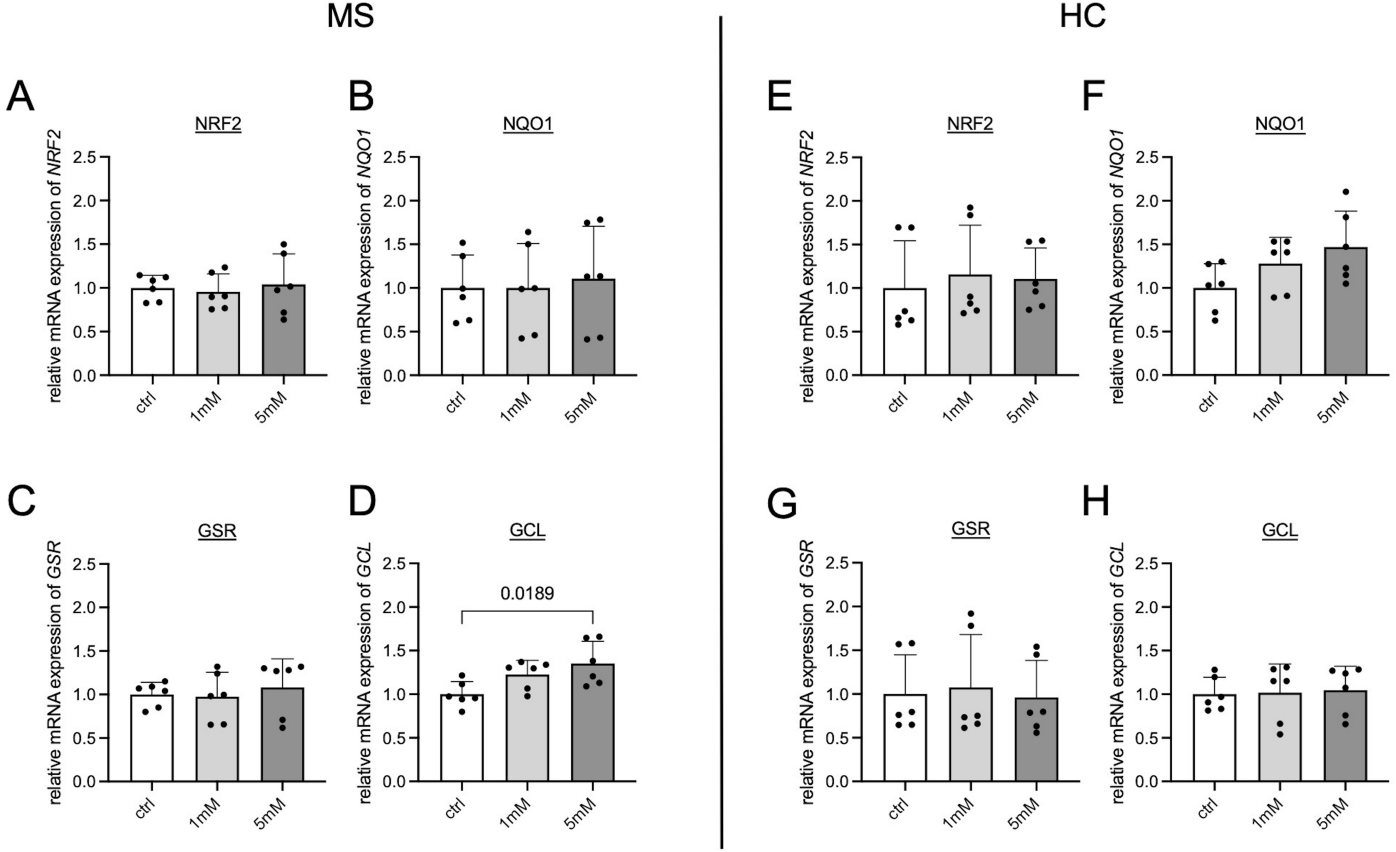
24-hour NALL treatment and oxidative stress regulator expression. MS-specific hiPNs showed no changes in NRF2 **(A)**, NQO1 **(B)** and GSR **(C)** expression after 24-hour NALL treatment, whereas significantly higher expression of GCL **(D)** was observed. In hiPNs from HCs, no significant changes were observed in any of these targets **(E–H)**. Data represented as mean ± SD. Statistics: Kruskal-Wallis with Dunn’s *post-hoc* test (N = 3, n=6). ctrl, control; GCL, glutamate-cysteine ligase; GSR, glutathione reductase; HC, healthy control; hiPNs, human induced primary neurons; MS, multiple sclerosis; NALL, N-acetyl-L-leucine; NRF2, nuclear factor (erythroid-derived 2)-like 1; NQO1, NAD(P)H:quinone oxidoreductase 1.

First, we treated hiPNs with NALL for 24 hours to investigate cellular behaviour under NALL treatment. We observed higher levels of the ratelimiting enzyme GCL in MS-specific hiPNs with 5 mM NALL (relative mRNA expression of GCL to control: 1 mM NALL 1.23±0.16; p= ns; 5 mM NALL 1.35±0.25; p= 0.0189; **Figure 6D**). However, we did not observe other effects of NALL in MS- or HC-specific hiPNs (**Figures 6A–C, E–H**).

### 3.5 MS- and HC-specific hiPNs react differently to induced stress by ST with inconsistent effects of NALL

To investigate resistance against induced oxidative stress, we examined two setups: hiPNs were treated with ST/NALL cotreatment for 6 hours or were pretreated with NALL for 24 hours with subsequent ST/NALL cotreatment for 6 hours.

**Figure 7:**
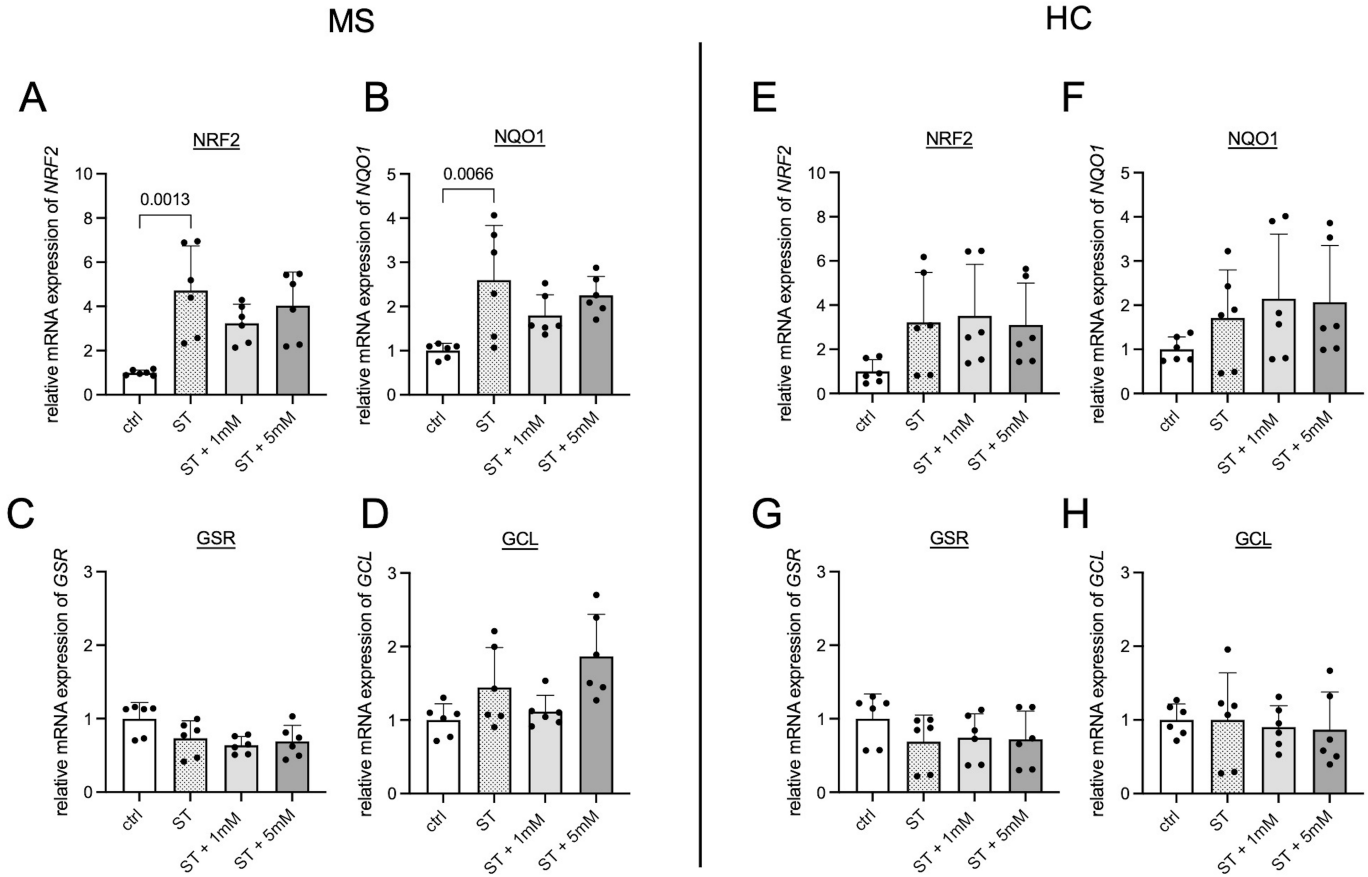
ST/NALL cotreatment for 6 hours indicated no effect of NALL on hiPNs during induced oxidative stress. NRF2 **(A)** and NQO1 **(B)** were significantly upregulated under ST treatment in MS-specific hiPNs, while no significant changes were observed for GSR **(C)** and GCL **(D)**. No significant changes could be observed in HC-specific hiPNs **(E–H)**. Data represented as mean ± SD. Statistics: Kruskal-Wallis with Dunn’s *post-hoc* test (N = 3, n=6). ctrl, control; GCL, glutamate-cysteine ligase; GSR, glutathione reductase; HC, healthy control; hiPNs, human induced primary neurons; MS, multiple sclerosis; NALL, N-acetyl-L-leucine; NRF2, nuclear factor (erythroid-derived 2)-like 1; NQO1, NAD(P)H:quinone oxidoreductase 1.

Our results showed significantly higher expression of NRF2 and NQO1 after a 6-hour ST treatment in MS-specific hiPNs (relative mRNA expression of NRF2 to control: ST 4.72±2.02; p= 0.0013; relative mRNA expression of NQO1 to control: ST 2.60±1.24; p= 0.0066, **Figures 7A, B**), but neither of GSR and GCL expression (**Figures 7C, D**) nor in response to NALL cotreatment (**Figures 7A–D**). HC-specific hiPNs demonstrated no significant changes in target expression (**Figures 7E–H**).

**Figure 8:**
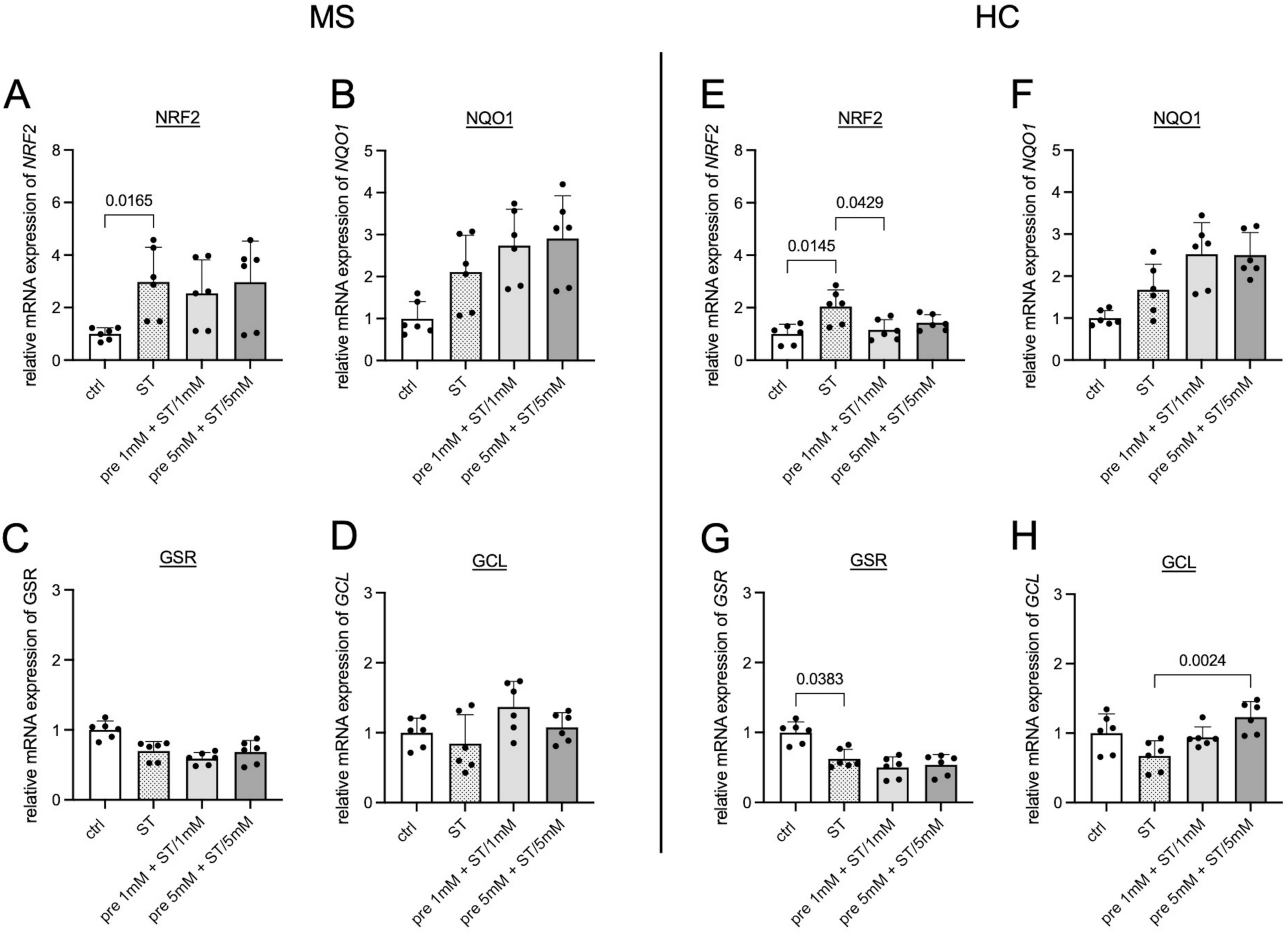
Pretreatment with NALL and subsequent ST/NALL treatment for 6 hours showed significant regulation of oxidative stress targets. NRF2 expression is upregulated in response to ST in both MS- and HC-specific hiPNs **(A, E)**, but only 1 mM NALL in HC-specific hiPNs downregulates NRF2 expression after NALL pretreatment during ST-induced stress **(E)**. MS-specific hiPNs showed no significant changes in NQO1, GSR and GCL expression **(B–D)**. In HC-specific hiPNs, no changes were observed in NQO1 expression **(F)**. Significantly lower expression of GSR was observed after ST, but no effect of NALL was observed **(G)**. Upregulated expression of GCL was observed after 5 mM NALL pretreatment compared to ST alone **(H)**. Data represented as mean ± SD. Statistics: Kruskal-Wallis with Dunn’s *post-hoc* test (N = 3, n=6). ctrl, control; GCL, glutamate-cysteine ligase; GSR, glutathione reductase; HC, healthy control; hiPNs, human induced primary neurons; MS, multiple sclerosis; NALL, N-acetyl-L-leucine; NRF2, nuclear factor (erythroid-derived 2)-like 1; NQO1, NAD(P)H:quinone oxidoreductase 1.

Pretreatment with NALL, followed by ST/NALL treatment, confirmed NRF2 upregulation in MS-specific hiPNs after ST treatment (relative mRNA expression of NRF2 to control: 2.97±1.33; p= 0.0165; **Figure 8A**), but not for NQO1 (relative mRNA expression of NQO1 to control: 2.11±0.87; p= ns; **Figure 8B**). Again, no changes in GSR or GCL expression or in response to NALL were observed (**Figures 8C, D**).

By contrast, hiPNs from HCs showed increased NRF2 response to ST alone not demonstrated in the previous experiment (relative mRNA expression of NRF2 to control: 2.05±0.63; p= 0.0145; **Figure 8E**) that is seemingly downregulated with 1 mM NALL (relative mRNA expression of NRF2 to ST: pre NALL/ST/1 mM NALL 1.15±0.39; p= 0.0429; pre NALL/ST/5 mM NALL 1.43±0.30; p= ns, **Figure 8E**).

Significantly lower GSR expression was detected in the ST group (relative mRNA expression of GSR to control: ST 0.62±0.13; p= 0.0383; **Figure 8G**). GCL expression was upregulated with the 5 mM NALL dosage (relative mRNA expression of GCL to ST: pre NALL/ST/5 mM NALL 1.23±0.22; p= 0.0024; **Figure 8H**). No changes were observed for NQO1 expression in HC-specific hiPNs (**Figure 8F**).

### 3.6 Pathway analysis demonstrates differential responses to induced stress of MS- and HC-specific cells

The final part of our study investigated potential pathway targets that could be involved in NALL effects on hiPNs.

We analyzed targets in the apoptotic signaling pathway and in the pathway responsible for cell growth and survival. BAD, BAX and BCL2 are all members of the BCL2 family, playing a key role in the intrinsic apoptosis pathway. BAX and BAD are pro-apoptotic, promoting mitochondrial permeabilization and sensitizing cells to apoptotic stimuli, whereas BCL2 is anti-apoptotic, protecting the cell from programmed cell death and preserving mitochondrial integrity (33, 34). The interaction between these targets determines whether a cell survives or dies under stressful conditions.

Conversely, we investigated targets that play a key role in regulating cell survival, growth, and metabolism. AKT3 is a serine/threonine kinase that promotes cell survival and growth by phosphorylating downstream targets such as mTOR, which regulates protein synthesis, energy metabolism and cell survival or growth (35). MAPK1 is an essential enzyme for RAS-MAPK signaling pathways, promoting cell growth, survival, differentiation and proliferation (36). The interaction between AKT/mTOR and MAPK1 integrates important functions such as growth factors and nutrient signaling, which are crucial for maintaining functional cell homeostasis and adaptation under stressful conditions.

**Figure 9:**
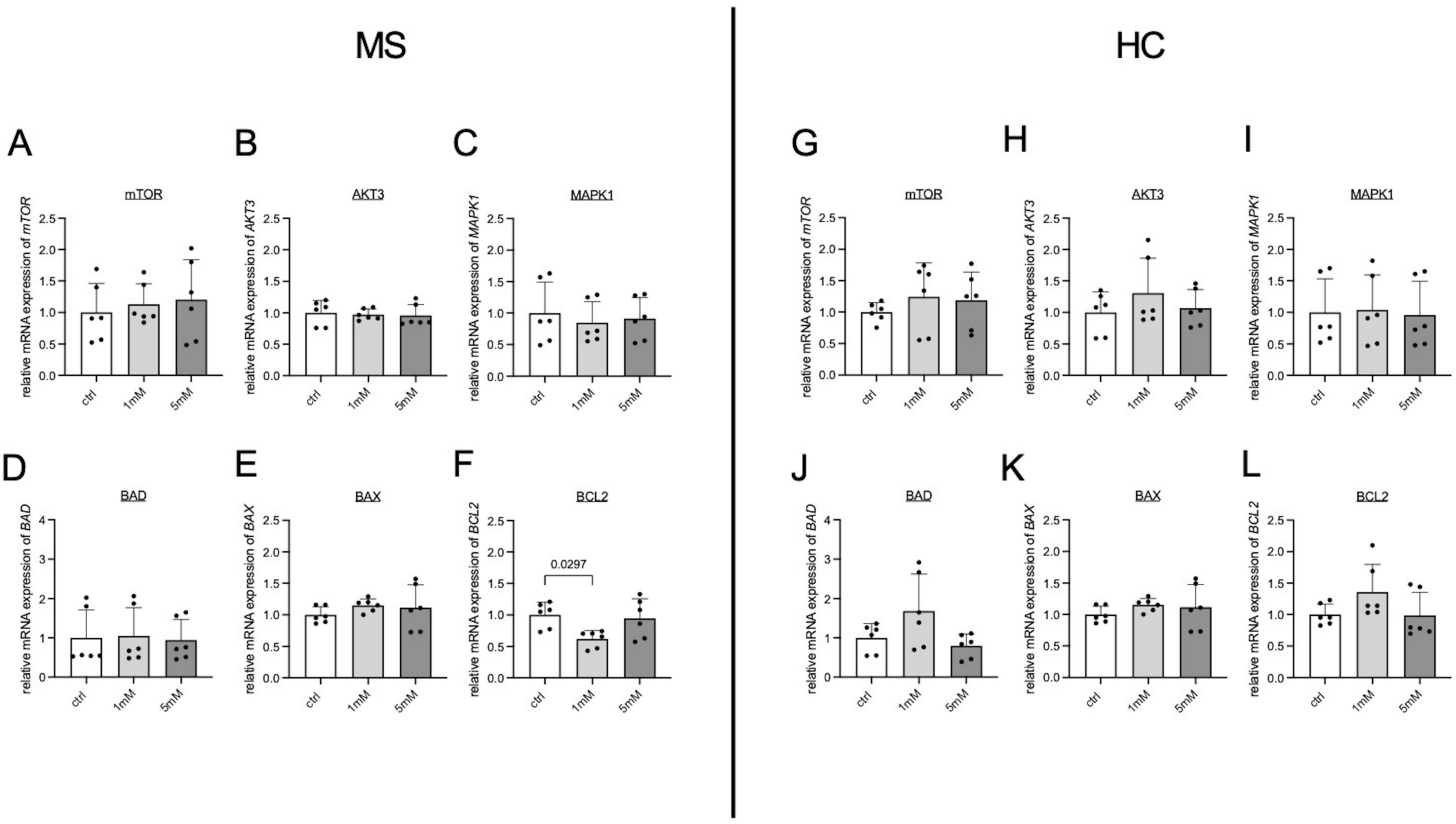
Pathway analysis after 24-hour NALL treatment. No differences were observed in MS- and HC-specific hiPNs in either survival **(A–C, G–I)** or apoptotic pathway targets **(D, E, J–L)**. However, a significant downregulation of BCL2 was observed in MS-specific hiPNs **(F)**. Data represented as mean ± SD. Statistics: Kruskal-Wallis with Dunn’s *post-hoc* test (N = 3, n=6). AKT3, proteinkinase B; BAD, BCL2 antagonist on cell death; BAX, BCL2 associated X protein; BCL2, B-cell lymphoma 2; ctrl, control; HC, healthy control; hiPNs, human induced primary neurons; MAPK1, mitogen-activated protein kinase 1; MS, multiple sclerosis; mTOR, mechanistic target of rapamycin; NALL, N-acetyl-L-leucine.

After 24-hour NALL treatment at 1 mM and 5 mM, we observed significant downregulation of BCL2 after 1mM NALL treatment (relative mRNA expression of BCL2 to control: 1mM NALL 0.62±0.1334; p= 0.0297; **Figure 9F**), but no significant changes in any other pathway targets in either MS- or HC-specific hiPNs (**Figures 9A–L**).

**Figure 10:**
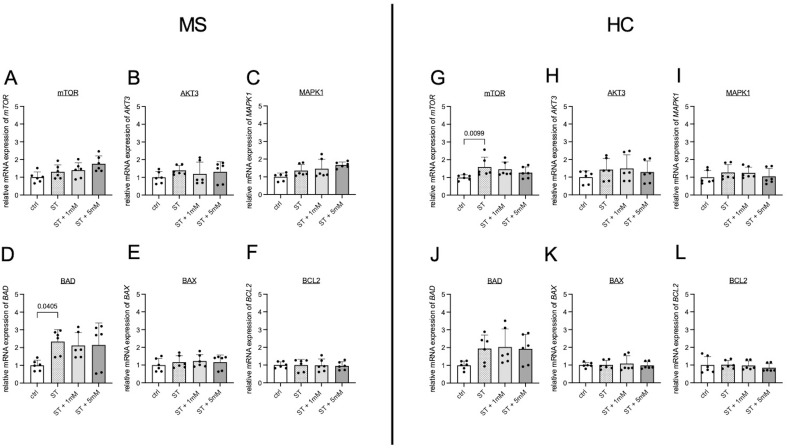
ST/NALL cotreatment showed no significant changes in pathway analysis. MS-specific hiPNs revealed significantly higher expression of BAD after ST exposition **(D)**, but no changes in other targets or in response to NALL **(A–C, E, F)**. HC-specific hiPNs showed significantly higher mTOR expression after ST treatment **(G)** and modest, but not significant BAD upregulation **(J)**. No other changes were observed **(H, I, K, L)**. Data represented as mean ± SD. Statistics: Kruskal-Wallis with Dunn’s *post-hoc* test (N = 3, n=6). AKT3, proteinkinase B; BAD, BCL2 antagonist on cell death; BAX, BCL2 associated X protein; BCL2, B-cell lymphoma 2; ctrl, control; HC, healthy control; hiPNs, human induced primary neurons; MAPK1, mitogen-activated protein kinase 1; MS, multiple sclerosis; mTOR, mechanistic target of rapamycin; NALL, N-acetyl-L-leucine.

Pathway target analysis in context of stress induction through ST and NALL cotreatment reveal that MS-specific hiPNs showed significantly higher expression of BAD in response to ST which is unaltered by NALL (relative mRNA expression of BAD to control: ST 2.34±0.68; p= 0.0405; **Figure 10D**). No other significant changes were observed. In HC-specific hiPNs, mTOR is significantly upregulated after ST, again unchanged by NALL cotreatment (relative mRNA expression of mTOR to control: ST 1.59±0.56; p= 0.0099; **Figure 10G**). BAD expression also increased in HC-specific hiPNs after ST, but did not reach statistical significance (relative mRNA expression of BAD to control: ST 1.94±0.76; p= 0.1430; **Figure 10J**). Other targets did not seem regulated in our experiments (**Figures 10A–C, E, F, H, I, K, L**).

**Figure 11:**
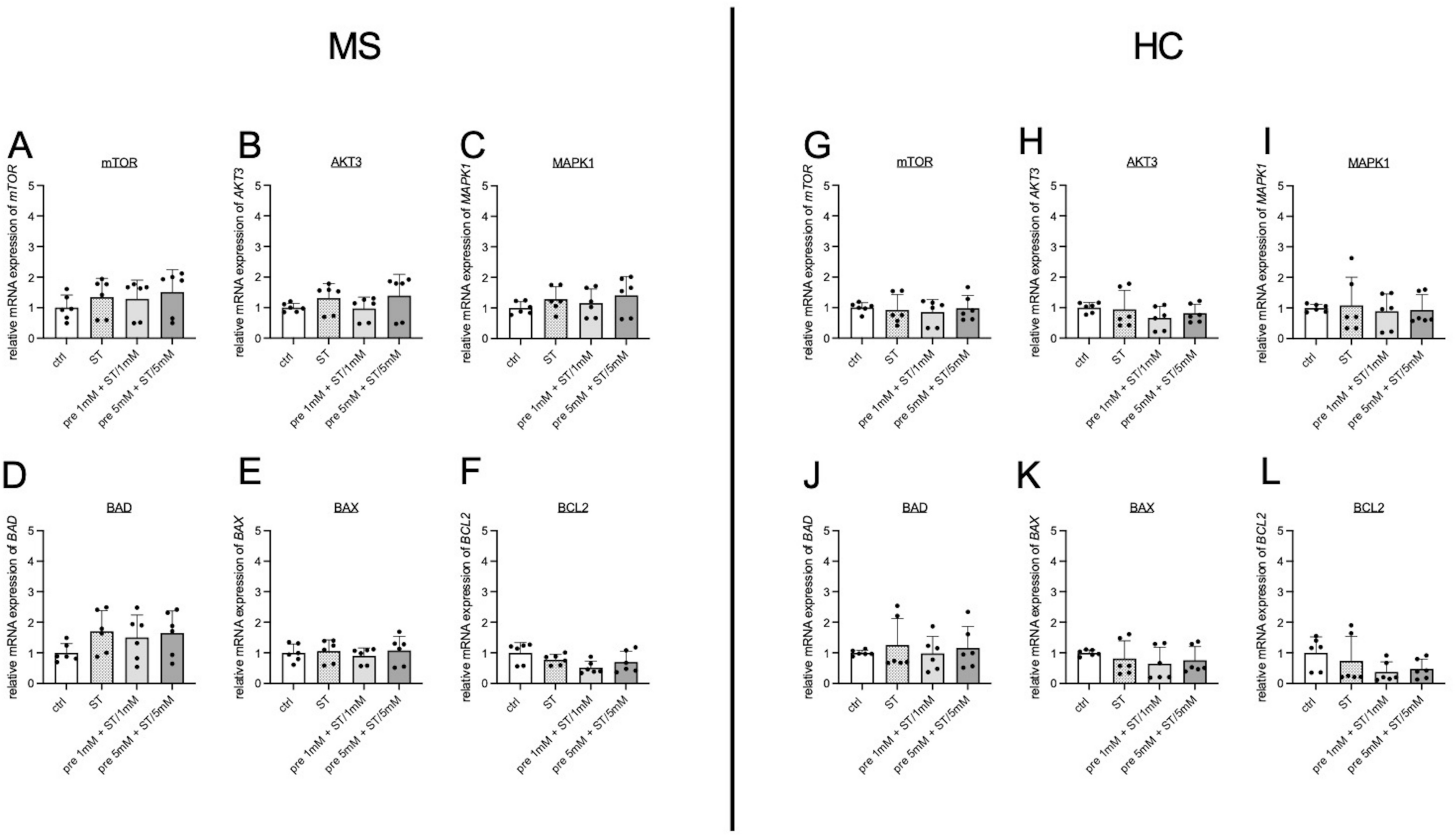
Pretreatment with NALL and subsequent ST/NALL cotreatment showed no changes in survival or apoptotic targets either in MS- **(A–F)** or HC- **(G–L)** specific hiPNs. Data represented as mean ± SD. Statistics: Kruskal-Wallis with Dunn’s *post-hoc* test (N = 3, n=6). AKT3, proteinkinase B; BAD, BCL2 antagonist on cell death; BAX, BCL2 associated X protein; BCL2, B-cell lymphoma 2; ctrl, control; HC, healthy control; hiPNs, human induced primary neurons; MAPK1, mitogen-activated protein kinase 1; MS, multiple sclerosis; mTOR, mechanistic target of rapamycin; NALL, N-acetyl-L-leucine.

In our last setup of pathway investigations hiPNs were pretreated with NALL for 24 hours with subsequent cotreatment of ST/NALL for 6 hours. Our results showed no significant changes in any pathway target in MS- and HC-specific hiPNs (**Figures 11A–L**). The ST groups did not demonstrate the changes shown before for mTOR and BAD (**Figures 11A, D, G, J**).

Taken together, our findings indicate variable mTOR and BAD responses to stress induction with ST, but no changes with exposure to NALL.

## 4 Discussion

MS is associated with irreversible neuronal loss and steady disease progression. Oxidative stress is considered as a key contributor to neurodegeneration, as it promotes mitochondrial dysfunction and exacerbates axonal injury (4, 37, 38). There is currently limited treatment for progressive components of the disease (39). Consequently, therapies targeting mechanisms besides autoimmune inflammation in the context of MS constitute a significant focus of current research.

We used an established model of hiPNs from MS- and HC-specific individuals generated from renal proximal tubule epithelial cells (23). We treated hiPNs with NALL alone, in cotreatment with different damaging substances as well as a pretreatment with NALL with subsequent damage.

Cellular functionality was analyzed via apoptosis and cell viability markers. Caspase 3 and 7 are executioner caspases that mediate the final steps of apoptosis and programmed cell death (26). ST is a protein kinase inhibitor that induces apoptosis via caspase-dependent and independent and mechanisms (40). Contrary to our hypothesis, we observed no changes in caspase 3/7 activity in either MS- or HC-specific hiPNs with NALL after stress induction with ST potentially indicating that NALL may not limit apoptosis rates after this particular stressor.

ATP is the main energy molecule of the cell. In neurons, it plays a key role in maintaining the membrane potential and transmitting signals and is primarily produced by mitochondria (41, 42). A study in a Parkinson’s disease model reveals that NALL is able to enhance ATP-linked respiration and maximum respiratory capacity (43). We observed significant downregulation of ATP levels in hiPNs from HC after 30-minutes and 1-hour 5 mM NALL treatment as a transient effect. Interestingly, previous studies suggest that NALL may not simply enhance ATP production but rather normalize cellular energy metabolism, indicating a biphasic mode of action (19). In this context, the observed reduction of ATP levels following NALL treatment may reflect a regulatory adjustment. In view of our other findings and lacking additional ATP reduction upon longer NALL exposure, we do not deem the transient ATP reduction solely in HC-derived cells as a sign of classical toxicity.

After ST-induced damage, results in MS- and HC-specific hiPNs were inconsistent. A reduction of ATP activity was only seen in HC-specific hiPNs after ST administration, whereas in MS-specific hiPNs, ST did not alter ATP activity. As only relative changes to control are measured, it remains speculative whether MS-specific cells may have impaired ATP activity even in an untreated stage. 5 mM NALL reduced ATP activity with and without NALL pretreatment after ST. While quantitatively, effects may appear small, they were still significant. Studies investigating metabolic differences in iPSC-derived astrocytes have found that MS-specific astrocytes exhibit some disease-specific features, such as impaired glutamate uptake and release, increased mitochondrial fission and increased oxidative stress (44). It stands to reason that such patterns could also appear in hiPNs. Translating this to our setting, we had expected lower ATP after ST damage that is potentially more pronounced in MS-specific hiPNs and may be restored with NALL. This could not be found.

NALL predominantly exerts neuroprotective effects, likely through stabilizing neuronal metabolism and membrane function rather than inducing neuroregenerative processes such as neurogenesis or axonal repair (13, 16). In our neurite analyses, we found that NALL did not affect regeneration following prior injury in MS and HC. However, we observed neuroprotective effects of NALL when co-administered during damage. Cotreatment with noco/NALL, resulted in significantly longer neurites in both MS-and HC-specific hiPNs compared to neurite lengths after nocodazole damage. These effects of NALL on neurite outgrowth have been demonstrated for the first time. Our literature search did not identify comparable data.

Previous studies have shown that NALL can act as an MCT1 substrate (15). The specific uptake of NALL via MCT1 as here firstly demonstrated in hiPNs suggests that the proposed transporter switch is functionally relevant in neurons. Blocking MCT1 should thus result in a reversion of the effects of NALL on neurite outgrowth. In fact, after MCT1 blockade at two different dosages of AZD, neurite lengths did not differ between the injury condition and the conditions with additional NALL administration in MS and HC cells supporting that the previously observed effects are dependent on intracellular NALL uptake.

Based on the findings in neurite outgrowth restoration by NALL, potential mechanisms in oxidative stress defense and pathways of the most important protective and survival targets were investigated. NALL treatment leads to significantly higher GCL expression in MS-specific hiPNs. GCL is the ratelimiting enzyme in glutathione synthesis (32). The presence of more GCL enables the synthesis of greater quantities of glutathione, thereby enhancing the ability of cells to counteract oxidative stress and other influences (45, 46). An impaired GCL activity is associated with reduced GSH levels that cannot be replenished by other GSH precursors (47). A study using an iPSC-based model demonstrated that elevated GSH levels and thus greater antioxidant capacity, provide better protection against oxidative stress (48). Increased GCL expression upon NALL treatment suggests enhanced glutathione biosynthesis capacity, which is a key determinant of neuronal resistance to oxidative stress. NALL may thus prepare cells for oxidative stress in a disease-specific setting, but not in cells of healthy donors. Donor-specific differences between neurons derived from disease vs. health states may require additional research.

To investigate whether MS-specific hiPNs are better protected against oxidative stress with NALL, ST-induced stress was elicited. Interestingly, ST only resulted in increased NRF2 and NQO1 expression in MS-specific hiPNs, potentially indicating that still, HC-specific hiPNs are more resilient to induced damage. Effects of NALL were not demonstrated in this cotreatment setting. NRF2 upregulation was robustly also shown in the pretreatment setting after ST-induced damage, this time in both MS and HC. In HC, GSR downregulation was additionally detected. NALL pretreatment only exhibited effects in hiPNs of healthy donors, namely reducing NRF2 expression and increasing GCL expression compared to ST damage alone. A clear dose dependency, however, was not detected. Effects of NALL to an extreme stressor as ST may thus only be detectable outside the disease stage. We have not investigated higher NALL dosages to analyze whether effects become apparent upon dose augmentation in MS-specific conditions. Studies on mice astrocytes reveal that preconditioning of NRF2 is related to increase of antioxidant targets and mechanisms (NQO1, GSH redox system) (49). While other studies also showed that NRF2 activation is associated with expression of antioxidant system targets (50). Although the precise molecular mechanism of NALL remains unclear, recent studies indicate that it has wide-ranging effects on mitochondrial, lysosomal, and synaptic signaling pathways (43, 51).

NALL exposure in the absence of the specific stressor ST downregulated BCL2 but only at 1mM NALL. Other than that we did neither observe altered pathway targets in MS- nor HC-specific conditions. However, upon ST treatment, MS-specific hiPNs reacted with an upregulation of pro-apoptotic BAD whereas HC-specific hiPNs upregulated mTOR. This underscores the differential response to stress in disease vs. healthy state. Increased mTOR expression has been associated with the cells’ attempts to activate survival-promoting signaling pathways in response to stress and apoptosis (52, 53). The mTOR signaling pathway needs good regulation due to the fact that hyperactivation of mTOR is associated with neuronal dysfunction. Normalized mTOR activity can restore neuronal homeostasis, improve stress resistance and prevent cell deficits (53–55). Effects of NALL were not detected in this setting.

As a proapoptotic target of the BCL-2 family, BAD is overexpressed in MS-specific hiPNs after ST treatment, and less prominent in HC, suggesting that hiPNs are more susceptible to apoptosis due to ST treatment particularly in MS, and NALL cannot compensate for this. However, we also observed that neither BCL-2 nor BAX are significantly altered in hiPNs after ST. BAD inhibits BCL-2, thereby inhibiting anti-apoptotic signals. However, BAX is the executor of apoptosis and becomes activated upon initiation of apoptotic signaling while its activity is antagonized by BCL-2 (56, 57), a process that remains unchanged in our setup. This leads us to assume that NALL cannot prevent the cell from becoming susceptible to apoptosis, but that it is also not fully executed after 6 hours of ST treatment.

After pretreatment with NALL, followed by stress induction, no significant changes were observed in any of the examined pathway targets in MS- or HC-specific hiPNs. Unfortunately, the ST-induced stress response (independent of NALL exposure) seemed to be less robust in this experimental setting. If any, a slight BAD signal to ST may be present in MS-specific hiPNs, corresponding to the previous setting.

The principle finding of this study is that NALL has neuroprotective effects on hiPN neurites during nocodazole-induced microtubule destabilization in both MS- and HC-derived neurons. This effect is dependent on transporter-mediated NALL uptake and is abrogated by MCT1 blockade.

Mechanistic analyses of oxidative stress markers and survival/apoptotic pathways were conducted using staurosporine, a kinase inhibitor that induces damage through mechanisms that are distinct from microtubule destabilization. Clear indications of involved pathways cannot be drawn from our data. We observed differential responses to NALL comparing MS- and HC-specific hiPNs in absence of a stressor and potentially higher vulnerability to damage in MS-specific hiPNs. A regulatory effect of NALL to induced oxidative stress was yet only detected in HC-specific hiPNs. These differences of neuronal behavior depending on health vs. disease state need further dedicated research.

In our research, we use a model of human-induced primary neurons. The limitations of this iPSC culture are that it is time- and resource-consuming and difficult to replicate due to variability between different donors that respond differently to various stimuli. Sample sizes are thus limited and further confirmation in independent settings is necessary. Although this non-invasive human model could help reducing animal experiments, broader generalizability is currently lacking. To enhance translational relevance of our findings, validation in independent settings and with larger cohorts is required. The inclusion of donors with progressive MS might provide additional insights. Nevertheless, this model provides a valuable proof-of-concept using human material potentiating a translation to the human situation.

Our aim was to investigate neuron-inherent reactions to induced damage comparing MS and HC. The use of artificial stressors in our experimental system induces a short-term, near-maximal stress response. While this provides a useful proof-of-concept and demonstrates that the observed effects can be detected under extreme conditions, these findings cannot be directly transferred to people with MS, in whom stress-related effects develop more subtle and over longer timescales. The culture system, however, would not allow for truly long-term exposure, and modelling of pleiotropic *in vivo* stress mechanisms will be challenging to achieve and to measure *in vitro*. We have thus decided to focus the experimental setting to well-established stress inductors and to investigate stress responses in absence of the inflammatory background that is present in *in vivo* as the latter may obscure more subtle neurodegenerative processes.

In line with the proof-of concept nature and previous preclinical research (19, 43), assumingly supraphysiological NALL concentrations have been applied in our experiments. Depending on body weight, oral daily NALL administration ranged from 2g to 4g in adult and pediatric populations with a favorable safety profile (20, 58). Pharmacokinetic studies have shown a steady state plasma concentration C_max_ of mean 8.3 (SD 3.3) µg/ml, corresponding to 48 ± 19 µM. Data on human brain concentrations of NALL are not available. However, pharmacokinetic studies on mice suggest that the drug is rapidly absorbed and distributed throughout the body following oral administration (59). Preclinical data thus support that NALL crosses the blood brain barrier and impacts neuronal circuits to elicit neuroprotective effects, including Niemann-Pick disease type C, GM2 gangliosidosis, and traumatic brain injury (19, 60). Clinical studies of Nieman-Pick disease type C have observed improvements of neurological function in a 30-month extension study (51). These results imply potential disease modifying properties, although the underlying mechanisms are not fully elucidated and NALL concentrations in the brain will be lower than applied in our system.

At the same time, plasma concentrations may not directly reflect intracellular concentrations, since MCT-mediated transport can result in the intracellular accumulation of NALL. Determining the physiologically relevant concentrations for mechanistic *in vitro* studies remains a significant challenge for future research (15, 19, 60).

Our work is exploratory and based on *in vitro* analyses. Clinical validation is lacking. As NALL has already been applied in individuals with Niemann-Pick disease type C and ataxia-teleangiectasia and effects on neurite outgrowth seemed robust over MS- and HC-specific cells in our experiments, we envisage using it as an add-on treatment of MS to potentially reduce neurodegeneration seems generally feasible. However, underlying mechanisms and adequate dosages still need to be determined for MS or other diseases, particularly as measurable effects of NALL pretreatment on oxidative stress were only detected in hiPNs of healthy donors. Our findings should be interpreted as reflecting direct neuronal responses rather than the complex disease environment present *in vivo*. A comprehensive evaluation of NALL in the context of MS will require models that consider inflammation, and the complex interactions with glial cells and other components of the neuroimmune axis. However, given the complexity of these processes, even advanced co-culture systems are unlikely to fully replicate the *in vivo* situation. Additional *in vivo* models may be necessary to evaluate the therapeutic potential and mechanisms of action of NALL in MS.

## Data Availability

The raw data supporting the conclusions of this article will be made available by the authors, without undue reservation.
