# Supplementary material for: Neuromodulatory effects of N-acetyl-L-leucine in a human induced neuronal cell culture system
Source: Front Immunol. 2026 Aug 5;17:1866462. doi: 10.3389/fimmu.2026.1866462 (PMC13485601; doi:10.3389/fimmu.2026.1866462)
Supplement: Supplementary file 1 [file DataSheet1.pdf]

Supplementary Material

1 Supplementary Figures and Tables

1.1 Supplementary Figures

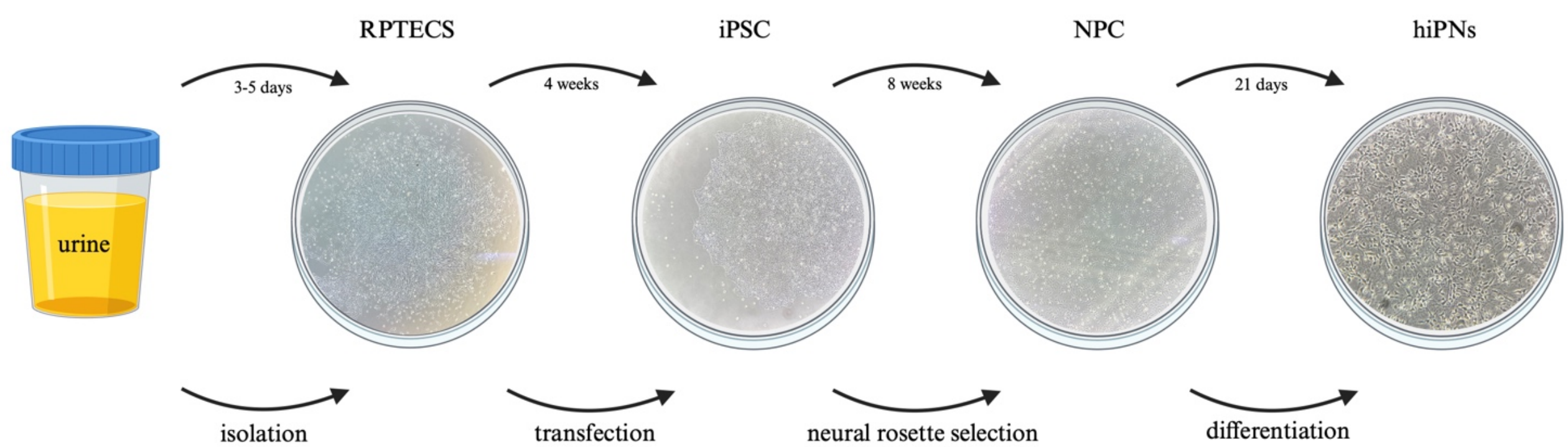

Supplementary Figure 1: Generation of human induced primary neurons. RPTECs were isolated from urine samples taken from patients with MS or HC. After 3–5 days, RPTEC colonies formed and were transferred after three passages. Transfection was performed via electroporation using episomal plasmids. Four weeks after transfection, colonies of iPSCs form. These can then be differentiated into embryoid bodies after four passages, which form neural rosettes. The rosettes are then selected and replated to produce NPCs. HiPNs can then be differentiated over a period of 21 days. Abbreviations: HC = healthy control; hiPNs = human induced primary neurons; iPSCs = induced pluripotent stem cells; MS = multiple sclerosis; NPCs = neural precursor cells; RPTECs = renal proximal tubule epithelial cells. Created in BioRender.

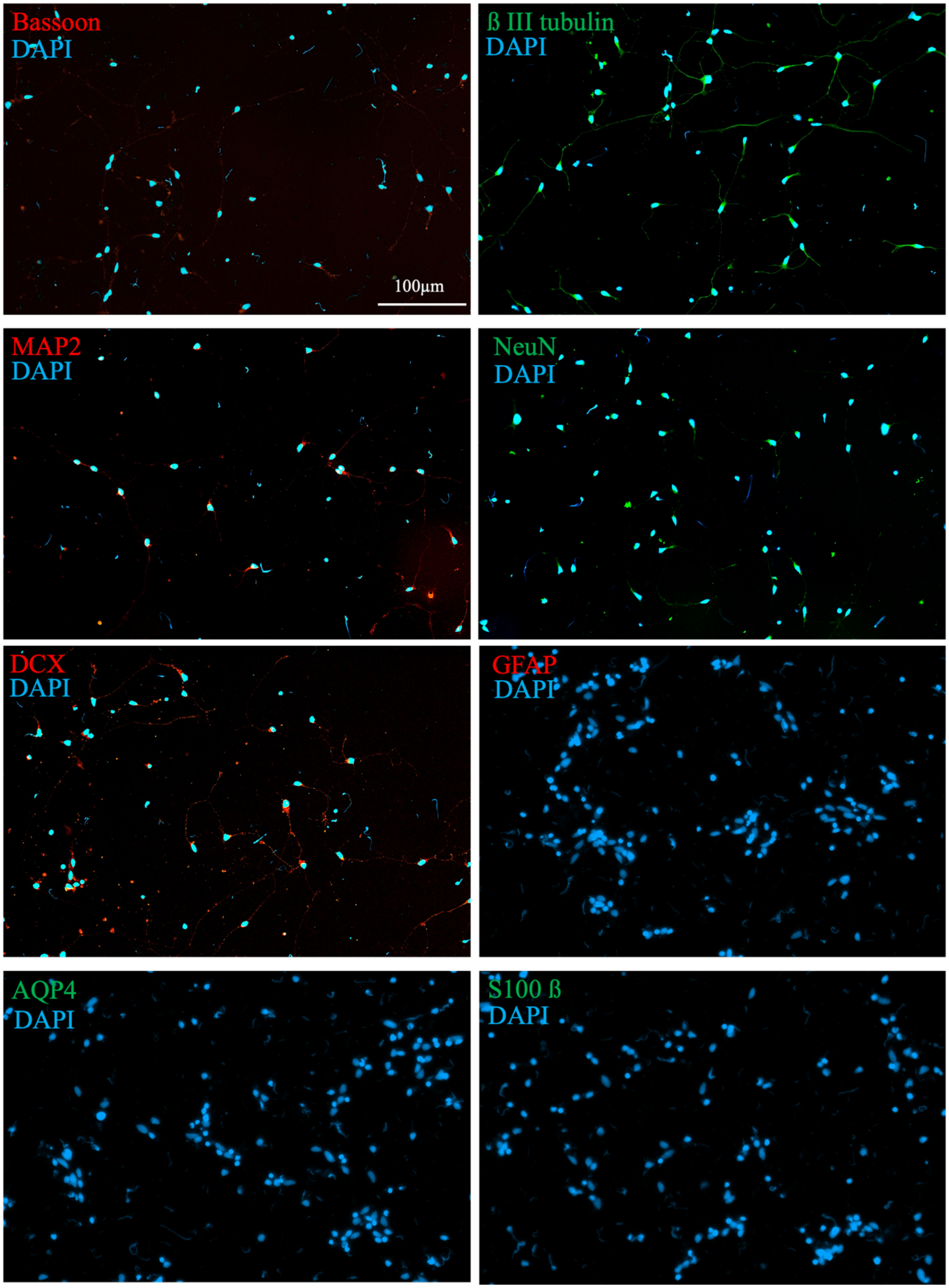

Supplementary Figure 2: Immunocytochemical validation of maturated human induced primary neurons on day 21 of differentiation. The figure shows various markers used to validate mature hiPNs. The images display the specific channel in red or green, as well as the cell nucleus stained with DAPI (blue). Bassoon - universal marker for synaptic terminals; Beta III tubulin - marker for neuronal

axons and neurites; MAP2 - neuronal cell bodies and dendrites; NeuN - cell nuclei; DCX - marker for immature neurons; GFAP - structural protein in astrocytes; AQP4 - transmembrane protein in astrocytes and S100 ß - calcium-binding protein in astrocytes. These exemplary images of the neuronal culture after differentiation, but prior to any treatment demonstrate presence of the neuronal markers and absence of astrocytic markers. Abbreviations: AQP4 = aquaporin 4; DAPI = 4',6-Diamidino-2-phenylindole; DCX = doublecortin; GFAP = glial fibrillary acidic protein; MAP2 = microtubule-associated protein 2; NeuN = neuronal nuclei antigen.

1.2 Supplementary Tables

Supplementary Table 1: Donor demographics of participants with MS and HC at the time of urine collection.

**Donors**	**MS**			**HC**		
	**1**	**2**	**3**	**1**	**2**	**3**
**Sex**	female	female	male	female	female	female
**Age (years)**	26	25	26	25	24	23
**Disease duration**	Initial diagnosis	Initial diagnosis	Initial diagnosis	-	-	-
**Disease-modifying therapy**	none	none	none	-	-	-

Abbreviations: HC = healthy control; MS = multiple sclerosis
